# The Use of Integrative Therapies in Patients with Amyotrophic Lateral Sclerosis in Shanghai, China

**DOI:** 10.1155/2013/613596

**Published:** 2013-12-02

**Authors:** Weidong Pan, Xiangjun Chen, Jie Bao, Yu Bai, Hua Lu, Qiudong Wang, Yi Liu, Canxing Yuan, Wenwei Li, Zhenguo Liu, Jun Liu, Xuying Zhu, Baofeng Qin, Dingfang Cai, Hua Zhou

**Affiliations:** ^1^Department of Neurology, Shuguang Hospital Affiliated to Shanghai University of Traditional Chinese Medicine, 528 Zhangheng Road, Pudong New Area, Shanghai 201203, China; ^2^Department of Neurology, Huashan Hospital Affiliated to Fudan University, Shanghai 200040, China; ^3^Department of Neurology, Putuo District Center Hospital Affiliated to Shanghai University of Traditional Chinese Medicine, Shanghai 200062, China; ^4^Center for Clinical Effectiveness Evaluation, Shuguang Hospital Affiliated to Shanghai University of Traditional Chinese Medicine, Shanghai 201203, China; ^5^Department of Neurology, Pudong New Area Hospital of Traditional Chinese Medicine, Shanghai 201200, China; ^6^Department of Neurology, Shanghai Hospital of Traditional Chinese Medicine, Shanghai 200071, China; ^7^Department of Neurology, Longhua Hospital Affiliated to Shanghai University of Traditional Chinese Medicine, Shanghai 200030, China; ^8^Laboratory for Neurology of Institute of Integrative Medicine, Zhongshan Hospital Affiliated to Fudan University, Shanghai 200093, China; ^9^Department of Neurology, Xinhua Hospital Affiliated to Shanghai Jiao Tong University, Shanghai 200092, China; ^10^Department of Neurology, Ruijin Hospital Affiliated to Shanghai Jiao Tong University, Shanghai 200025, China; ^11^Department of Cardiology, Shuguang Hospital Affiliated to Shanghai University of Traditional Chinese Medicine, Shanghai 201203, China

## Abstract

*Objective*. To investigate the current use of integrative therapies (IT) in the treatment of patients with amyotrophic lateral sclerosis (ALS). *Methods*. A cross-sectional, multicenter clinical epidemiological survey was conducted in 12 hospitals in Shanghai. We investigated the type and frequency of IT use and determined whether the use of IT correlated with demographic, social, or disease-specific characteristics in our patient population. *Results*. A total of 231 (89.5%) of 258 patients with ALS were eligible for the study and 229 (99% of all) of 231 reported the use of at least one IT for the treatment of ALS. Vitamins and Chinese herb decoctions, Chinese herb compounds, massage therapy, and acupuncture were the 5 most commonly used therapies. There was a strong association between education level, income, and use of IT. A household income of more than 75,000 RMB ($49,995) correlated with multiple IT use, and married patients used IT more often than single individuals. The main reasons for using IT were to treat weakness and fatigue, muscle atrophy, the development of ALS, depression, insomnia, limb pain or numbness, and side effects associated with Riluzole. *Conclusion*. The use of IT is common in patients with ALS in Shanghai. Vitamins and TCM are the most used additional therapies and the widespread and largely unexamined use of IT for ALS requires more attention.

## 1. Introduction

Amyotrophic lateral sclerosis (ALS), also known as Lou Gehrig's disease, is a relatively rare, adult-onset, rapidly progressive, and fatal disease that involves degeneration of upper and lower motor neurons [1]. Individuals with ALS most commonly die of respiratory failure or pneumonia within 2–5 years of diagnosis. There is currently no effective treatment for ALS. Riluzole, the current standard of care for ALS, only extends lifespan by 2-3 months and has undesirable side effects such as nausea and fatigue [2]. Integrative therapy (IT), as a holistic medical concept, might make contributions in boosting medical advances and safeguarding human health [3, 4]. Many neurologists attempt to identify effective treatments as IT in order to deal with various symptoms in ALS patients and to improve the activities of daily living (ADL) of the patient. The current study investigated the use of IT in patients with ALS in 12 hospitals in Shanghai, China, to determine the prevalence and spectrum of IT use by patients with ALS and to determine whether the use of integrative administration correlates with demographic, social, or disease-specific characteristics.

## 2. Materials and Methods

### 2.1. Subjects

Data were collected by interview from 231 patients with ALS treated in 12 hospitals, including 7 Western medicine hospitals and 5 integrative medicine hospitals, in Shanghai over a 6-month period (between December 1, 2012, to May 31, 2013). Patients with definite or probable sporadic ALS, according to the revised El Escorial criteria [5], were enrolled. The ages of the patients ranged from 20 to 80 years. Patients with dementia were excluded. The Shuguang Hospital Affiliated to Shanghai University of Traditional Chinese Medicine approved the protocol, and all participants provided informed consent.

### 2.2. Survey Tool

The patients were asked to fill out a structured questionnaire (original questionnaire printed in Chinese) that included demographic information, previous and current use of Western medicine, use of IT such as Chinese herbal decoctions, Chinese herbal compounds, acupuncture or acupressure and moxibustion, massage therapy, wild Jinsheng and Chinese caterpillar fungus (these 6 therapies are also defined as traditional Chinese medicine, TCM), Ginkgo biloba, vitamins, nutritional supplements, art therapy, magnets, music therapy, energy healing, homeopathy, chiropractic techniques, reflexology, relaxation techniques, spiritual healing, imagery, biofeedback, hypnosis supplements, psychotherapy, melatonin, fatty acids from fish oil concentrate, lipoic acid, Qi Gong exercise, Tai Chi quan, and antidepressant or other additional therapies. If a subject did not understand an item on the list, a standardized explanation of the therapy was provided. When a patient or caregiver/relative responded affirmatively to the use of one or more therapies, they were further asked about the frequency and duration of its use. We also asked how they learned about such therapy and whether the physician treating their ALS was consulted before initiating its use. We considered the use of vitamin E to be an IT because it has been proven not to be of use in the treatment of ALS.

ALS-related symptoms that have been treated were also evaluated, and the rationale for using IT was calculated for evaluation. The questionnaire forms were filled out by the patients or a caregiver (spouse or family member) in the presence of the physician and interviewers. Twelve specially trained investigators performed all assessments as interviewers. One investigator (P.R.R.) conducted all interviews, which ranged from 7 minutes to 1 hour depending on the extent of IT use.

In addition to the use of IT, the patients were also asked about their marital status, household income, and education level. Additional data on each patient included the age at onset of ALS, its duration, and any surgical procedures for ALS. The ALS functional rating scale revised (ALSFRS-R) total score [6] was also used to evaluate the clinical severity of the disease.

### 2.3. Statistical Methods

EpiData software (version 3.2) was used for data entry and data documentation. The original data were converted to the SAS system (version 9.2) for statistical analysis [7]. All data are expressed as the mean ± SD. The rate or constituent ratio was calculated for categorical variables. The independent samples *t*-test was used to compare the means between males and females and the chi-square test was used to compare the constituent ratio between the two groups. A significant difference was defined as *P* < 0.05.

## 3. Results

### 3.1. Patient Characteristics

Initially, 258 subjects (patients or their spouse or relative) were invited to fill out the questionnaires with their physicians and the interviewers in the 12 hospitals. Of these 258 patients, 231 (89.5% overall response rate, age 62.8 ± 14.7 years, mean ± SD) were eligible for the study and completed the interview. A total of 27 patients were excluded: 16 because they refused to provide information about their additional treatments and (or) households, 5 who were diagnosed with dementia, and 6 because they could not be diagnosed as having definite or probable sporadic ALS. Otherwise, the data set was complete. The characteristics of the 231 patients are shown in Table 1. There were 148 male patients (64%, 57.2 ± 15.6 years) and 83 (36%, 64.9 ± 17.3 years) female patients. The percentage of respondents 40 years of age or older (212/231, 135 males and 77 females) were 91.77% and 8.33% in those younger than 40 years of age (13 males and 6 females). The mean postelementary education level was 6.8 ± 3.9 years for all eligible subjects (7.3 ± 3.1 years for males and 5.6 ± 3.3 years for females).

### 3.2. Use of IT

**Figure 1:**
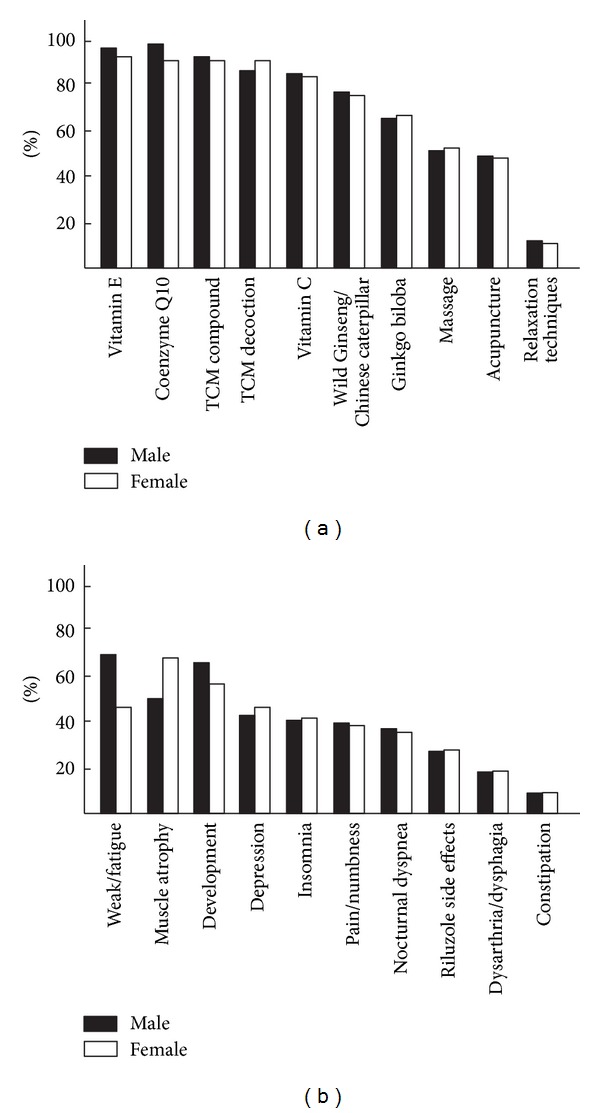
Integrative therapy (IT) usage rates and the reasons for using IT. Demonstrates the usage rates (a) of integrative medicine (IT) and the reasons (b) of using IT in treating for patients with amyotrophic lateral sclerosis (ALS) in Shanghai, China.

A total of 229 (99%) patients reported the use of at least one IT for the treatment of ALS; 96% reported using two therapies and 87% reported using more than two. Specifically, 47% used 5 or more therapies. Vitamins and Chinese herbal decoctions, Chinese herbal compounds, massage therapy, and acupuncture were the 5 most commonly used therapies. Both vitamins and Chinese herbal decoctions and/or Chinese herbal compounds were used by 209 patients (90.5%). The most common were vitamin E (95%) at an average daily dosage of 433.14 ± 138.29 IU (range, 400–2,400 IU), coenzyme Q10 (95.47%) with a mean daily dosage of 48.6 ± 27.48 mg (range, 30–240 mg), Chinese herbal decoctions (90.6%), Chinese herbal compounds (94.8%), and multivitamins (91%), followed by vitamin C (86.69%), wild Ginseng and Chinese caterpillar fungus (76.90%), and Ginkgo biloba (67%) (Figure 1(a)). Other IT used were massage therapy (56.83%), acupuncture (51.32%), relaxation techniques (16.44%), fatty acids from fish oil concentrate (15.92%), and magnets (5.27%). Most of the patients learned about IT from a family member or friend (68.11%), followed by the media (53.34%) and a healthcare professional (35.69%). The Internet was the referral source for 66.37% of patients or caregivers. Most patients (89.03%) did not use IT before the diagnosis of ALS. More than two-thirds (78.19%) of IT users did not consult their physician before starting IT. Among the demographic factors, gender was not correlated with the use of IT. Among the social variables, there was a strong association between education level, income, and use of IT. Patients with a college education or beyond used more IT (*r* = 0.799, *P* = 0.048). A household income of more than 75,000 RMB ($49,995) was also correlated with multiple IT use (*r* = 0.683, *P* = 0.042). Married patients used IT more often than unmarried individuals (*P* = 0.005). After dividing the patients into a TCM group and non-TCM group, those who used TCM had a higher daily dosage of Riluzole compared with nonusers (*P* = 0.004), but there was no difference in the ALSFRS score between the two groups. IT use was not correlated with duration of ALS, duration of diagnosis, or duration of treatment with Riluzole for ALS.

### 3.3. Reasons for Using Integrative Therapies

The most common reason for using IT was to treat weakness and fatigue (68.72% of male IT users) in males and muscle atrophy (66.35% of female IT users) in females (Figure 1(b)). The second most common reason for using IT was to delay the development of ALS (62.91% for males and 57.20% for females). The third was to treat atrophy (49.46%) in males and weakness and fatigue in females (44.87%). The other reasons for using IT were to deal with depression (43.66%), insomnia (42.26%), limb pain or numbness (40.7%), nocturnal dyspnea (39.4%), side effects of Riluzole (27.7%), dysarthria or dysphagia (19.74%), constipation (13.87%), poor appetite (12.63%), spasticity (12.19%), and abnormal sweating (6.98%). For the first 6 reasons (except the second), TCM (98.61% of these patients) was chosen to treat the symptoms (Figure 1(b)). No difference was found between males and females with respect to the use of TCM. Vitamin E (95.80%) was used most often to delay the development of ALS.

### 3.4. Use of Traditional Chinese Medicine

Various TCM compounds were used to treat patients with ALS. The most frequently used compounds were* Jinkui Shenqi pills* (13.09% of all TCM compound users), *Buzhong Yiqi pills* (11.32%), *Jianpi pills *(9.29%), *Yangxue Qingnao granules* (7.15%), *Congrong Tongbian Oral Liquid* (5.86%), and* Baohe pills* (3.89%). The most frequently used decoctions of traditional Chinese herbs by patients with ALS were *Sijunzi decoction* (14.70% of all TCM decoction users), *Bazhen decoction* (12.34%), *Shiquan Dabu decoction* (8.31%), *Buzhong Yiqi decoction* (6.68%), *Tianwang Buxin Dan* (5.52%), and *Guipi decoction* (4.98%). The Chinese herbal medicines (both decoctions and compounds) that were used most frequently were *Rhizoma atractylodis macrocephalae* (17.37% of both decoctions and compounds), *Codonopsis pilosula* (14.66%), *Radix pseudostellariae* (9.89%), *Eucommia ulmoides* (6.78%), *Radix achyranthis bidentatae* (6.19%), *Semen coicis* (5.28%), *Radices sileris* (4.74), *poria cocos* (3.82%), and *atractylis ovata* (3.16%).

Acupuncture therapy was often used to treat patients with ALS. The most frequently used acupoints were* Guanyuan *(CV4, 12.87% of all acupoints)*, Qihai* (CV6, 10.70%)*, Zusanli* (ST36, 9.97%), *Shenshu *(BL23, 9.69%), *Baihui *(GV20, 8.73%), *Sanyinjiao* (SP6, 7.92%), *Zhongwan* (RN12, 7.66%), *Hegu* (LI4, 7.50%), *Dazhui *(DU14, 6.65%), *Quchi* (LI11, 5.28%), *Taixi* (KI3, 4.77%), and* Neiguan* (PC6, 3.79%).

### 3.5. Subjective Effects of the Integrative Therapies

Approximately two-thirds (63.23%) of the patients reported no obvious effects from the IT therapies, while 24.69% indicated the IT was working for the symptoms but they did not feel the additional treatments resulted in any significant improvement. Only a few subjects (9.37%) reported they could obtain additional effects from IT therapies; the others were not able to evaluate the effects of IT therapies. Most of the effects of the IT therapies were improvements in subjective symptoms, such as feeling more comfortable, slightly happier, more energetic, experiencing better relaxful sleep or deeper sleep, better appetite, and even delayed development of ALS; however, no evidence-based study has been conducted.

### 3.6. Cost of Integrative Therapies

The mean cost of IT for all IT users was 1669 RMB ($270) per month. The most expensive was 6000 RMB ($595) per month, while the cheapest was 200 RMB ($33) per month. As of the time of this study, the mean total cost was 20,677 RMB ($3,434) for all IT treatments. The most spent was 53,275 RMB ($8,850) over 29 months, and the least spent was 8,360 RMB ($1,389) over 5 months.

## 4. Discussion

Identifying effective treatments for ALS is an important task for neurologists. IT might be one choice for treating various symptoms of ALS due to the benefits that have been reported in clinical studies [8–10]. We found that, among all respondents with ALS, there were more males than females, and most were 40 years old or older (Table 1). At least one IT was used by 99% of these patients and 96% used more than two therapies.

One reason for using IT might be due to the insufficiency of ALS therapies. There is currently no effective therapy for treating the symptoms and development of ALS. There is one drug, Riluzole; however, its efficacy is poor. Patients and their caregivers are desperately hoping for the development of an effective therapy, no matter what the method is. In the present study, we found that starting IT may not have been recommended by their physicians. Family members, friends, and even patients themselves often try to find effective methods to improve symptoms or delay the development of ALS. We found a strong correlation between IT use and higher levels of education and income. Patients or family members with high education level might gain more personal insight into the IT market easily, and they might have more payment capacity to pay the extra charges because the higher income reflects the fact that most IT are out-of-pocket expenditures. Married subjects used IT more often than singles because of their higher levels of education and income, and family members might have been more able to obtain information about treatment methods more easily than single subjects.

The IT used most often were vitamin E and TCM decoction or compounds (about 90.5% of IT users). The reasons for this may be as follows. First, vitamin E is often introduced as a complementary therapy for neurodegenerative disease [11], may function to prevent or delay senility [12–14], and is very cheap (200 UI × 100 pills = about 8–20 RMB ($1.33–3.32)) in China. Second, it has almost no side effects in the general Chinese population and is available over-the-counter (OTC). Furthermore, elderly people like to take it if they are feeling weak or tired. Many of these therapies are used without the knowledge of the treating physician. Third, TCM decoctions and compounds have proven effects on Chinese people and a long history of use in China, and they believe TCM decoctions and compounds might improve any disease. Furthermore, compared with the cost of taking other IT and Riluzole, vitamin E and TCM decoctions and compounds are much cheaper. From January 2012 to May 2013 in China, the mean price of TCM decoctions and compounds was about 10.23 to 72.87 RMB/per day, while *wild Ginseng* is 92.36 RMB/per day, Chinese caterpillar fungus supplements range from 190.28 to 365.37 RMB/per day, and Riluzole costs 160 RMB/per day (exchange rate; approximately 6 yuan/US dollar). Therefore, TCM decoctions and compounds are superior to Riluzole from a price perspective.

The most debilitating symptoms of ALS are weakness and fatigue, and with the development of ALS, atrophy becomes more grievous for the patient. However, no therapy is effective at treating these symptoms. Interestingly, the fourth most common reason for using IT was to treat the side effects of Riluzole, suggesting that Riluzole has more side effects than IT. Even though no evidence-based research has definitively identified the effects of IT in the treatment of ALS, family members, friends, and some physicians still want to try IT for serious problems with the hope it will be effective for treating the symptoms. We found no correlation between the use of IT and disease severity, as measured by the ALSFRS-R scores. This suggests that patients with ALS are not turning to IT in desperation. This finding may be disease-specific because the most common reasons cited by patients with brain tumors for the use of IT were the limited effectiveness of conventional therapies [15]. The strong correlation between IT use and higher levels of education and income demonstrate that most IT are out-of-pocket expenditures.

TCM decoctions and compounds seem to be chosen by most patients. The most cited TCM decoctions and compounds are *Sijunzi decoction* or *Jinkui Shenqi pills*, *Buzhong Yiqi pills*, and *Jianpi pills* or single herbs like *Panax Ginseng*, *Codonopsis pilosula*, *Astragalus mongholicus, Rhizoma Atractylodis Macrocephalae, Angelica sinensis, Poria cocos, Glycyrrhiza*, *Radix pseudostellariae*, and *Herba cistanche*, which are famous for increasing immunity and physical fitness in TCM [16–18] and according to TCM theory can “nourish the spleen and enrich vitality” which meet the TCM guideline in treating weakness and atrophy, and has been identified from other studies [8, 19, 20]. Most used acupoints also could “nourish spleen and enrich vitality” too in TCM theory.

The present results indicate that a high proportion of the ALS patients responding to this questionnaire did or would use IT without informing their physician, indicating that patients and physicians are not communicating effectively concerning the use of IT and that most patients are motivated to use IT by family, friends, and the media. In addition to improving communication between physicians and patients about IT, this survey, although limited, has provided an insight into IT utilization and referral patterns in ALS patients in Shanghai. Further investigation is needed to identify and quantify IT offered to ALS patients and the decision-making criteria used. The extensive and expanding use of IT requires further examination of their safety, efficacy, and drug interactions, as well as the factors that lead patients to use it. Physicians might include IT in their clinical practice when treating ALS patients.

## Figures and Tables

**Table 1:** Characteristics of the study population (*n* = 231).

Characteristic	Value
Mean age, *y* (range)	63.2 (29–76)
Sex, *n* (%)	
Male	148 (64%)
Female	83 (36%)
Average age at onset, *y* (range)	59.3 (28–75)
Mean duration of ALS, *y* (range)	2.1 (0.9–4.2)
Mean duration of diagnosis, *y* (range)	1.3 (0.3–3.6)
Mean ALSFRS-R score (range)	40.2 (28.3–44.8)
Marital status, *n* (%)	
Married	179 (77.5)
Single, divorced, or widowed	52 (22.5)
Education level, *n* (%)	
Less than high school	23 (10)
High school	96 (41.6)
Some college	58 (25.1)
College graduate	42 (18.2)
Graduate school	12 (5.2)
Household income, *n* (%)	
<$1,500	19 (8.2)
$1,500–3,000	23 (10)
$3,000–4,500	78 (33.8)
$4,500–6,000	45 (19.5)
$6,000–7,500	32 (13.8)
>$7,5000	34 (14.7)

ALS: amyotrophic lateral sclerosis; ALSFRS-R: amyotrophic lateral sclerosis functional rating scale revised.
